# Widespread promoter-mediated coordination of transcription and mRNA degradation

**DOI:** 10.1186/gb-2012-13-12-r114

**Published:** 2012-12-13

**Authors:** Mally Dori-Bachash, Ophir Shalem, Yair S Manor, Yitzhak Pilpel, Itay Tirosh

**Affiliations:** 1 Department of Molecular Genetics, Weizmann Institute of Science, 234 Herzl Street, Rehovot 76100, Israel; 2 Current address: Broad Institute of MIT and Harvard, 7 Cambridge Center, Cambridge, Massachusetts 02142, USA

## Abstract

**Background:**

Previous work showed that mRNA degradation is coordinated with transcription in yeast, and in several genes the control of mRNA degradation was linked to promoter elements through two different mechanisms. Here we show at the genomic scale that the coordination of transcription and mRNA degradation is promoter-dependent in yeast and is also observed in humans.

**Results:**

We first demonstrate that swapping upstream *cis*-regulatory sequences between two yeast species affects both transcription and mRNA degradation and suggest that while some *cis*-regulatory elements control either transcription or degradation, multiple other elements enhance both processes. Second, we show that adjacent yeast genes that share a promoter (through divergent orientation) have increased similarity in their patterns of mRNA degradation, providing independent evidence for the promoter-mediated coupling of transcription to mRNA degradation. Finally, analysis of the differences in mRNA degradation rates between mammalian cell types or mammalian species suggests a similar coordination between transcription and mRNA degradation in humans.

**Conclusions:**

Our results extend previous studies and suggest a pervasive promoter-mediated coordination between transcription and mRNA degradation in yeast. The diverse genes and regulatory elements associated with this coordination suggest that it is generated by a global mechanism of gene regulation and modulated by gene-specific mechanisms. The observation of a similar coupling in mammals raises the possibility that coupling of transcription and mRNA degradation may reflect an evolutionarily conserved phenomenon in gene regulation.

## Background

Gene expression patterns are regulated by a multitude of processes, and while these processes have traditionally been studied in isolation, numerous instances of cross-talk between them have been revealed in recent years [1-5]. These cross-talk events influence the efficiency and dynamics of gene regulation and reflect the high inter-connectivity between regulators of distinct processes. While initial studies focused on the cross-talk between successive processes that occur in the same cellular compartment (for example, co-transcriptional mRNA processing), recent evidence points to coupling between transcriptional events in the nucleus and mRNA regulation in the cytoplasm. For example, Rpb4/7 binds to mRNAs co-transcriptionally and shuttles with the mRNA to the cytoplasm where it influences mRNA degradation and translation [6-8]. Large-scale analyses in yeast have demonstrated a global coordination between transcription and mRNA degradation in response to environmental [9,10] and genetic [11] perturbations and during evolution [12], suggesting that the mechanistic coupling through Rpb4/7 and other components has a global impact on gene regulation.

Coupling of transcriptional induction with enhanced mRNA degradation generates a peaked response that is an important feature of dynamic biological systems that must respond quickly but transiently as they adapt to environmental changes [10,13,14]. However, we recently demonstrated, through comparison of yeast species grown in rich media [12], that this coupling is also evident in the steady-state regulation of gene expression. Thus, it is possible that the transcription-degradation coupling has evolved in order to facilitate peaked responses but subsequently became a fundamental aspect of regulation. The presence of this global coupling in other species (besides yeast) remains unclear.

Evolutionary changes in transcription and mRNA degradation were coupled both through *trans *and through *cis *mutations, as determined from analysis of interspecific hybrids [12]. Coupling through *trans *mutations presumably involves changes in the activity of protein complexes that regulate both transcription and degradation, such as Rpb4/7 and Ccr4-Not [15]. Coupling by *cis*-mutations was observed at more than a hundred genes, but the mechanism of *cis*-coupling mutations is unknown. We speculated that promoter *cis *mutations could affect the recruitment of transcription complexes that not only regulate transcription but also prime the newly transcribed mRNA for future regulation in the cytoplasm [16]. Interestingly, two recent studies demonstrated promoter-mediated effects on mRNA degradation through binding of Rap1 or through cell-cycle regulators [17,18]. Yet these specific mechanisms are unlikely to account for our previous observations [12] since the *cis*-coupled genes we discovered were underrepresented with Rap1 targets and with cell-cycle periodic genes (not shown). This suggests the existence of additional unknown coupling mechanism(s) through *cis*-regulatory sequences, which may involve promoter, UTR or open reading frame elements.

Here, we validate our previous prediction of *cis*-dependent coupling by swapping upstream *cis*-regulatory sequences (promoters and 5' UTRs) between *Saccharomyces cerevisiae *and *Saccharomyces paradoxus *and measuring the effect of swapping on mRNA levels and mRNA degradation. We further demonstrate the role of promoters in controlling mRNA degradation by showing that yeast genes that share a promoter tend to have similar mRNA degradation profiles. Based on these results we propose that a general promoter-mediated mechanism couples transcription to mRNA degradation of a large fraction of yeast genes and that this mechanism may be modulated by various *cis *and *trans *elements. Finally, we present evidence for a similar coupling in mammalians through analysis of differences in mRNA degradation between different cell types or species.

## Results

### Swapping orthologous sequences reproduces interspecies expression divergence and transcription-degradation coupling

**Figure 1:**
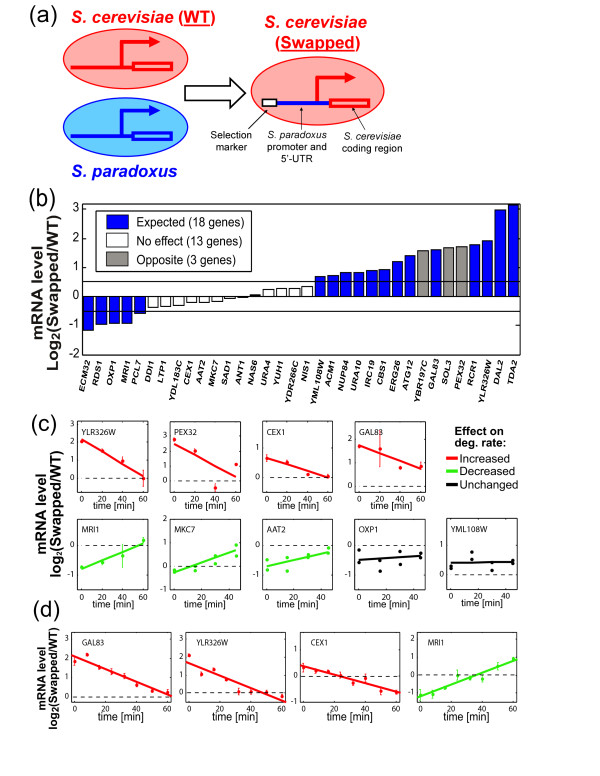
**Swapping of upstream regulatory sequences affects transcription and mRNA degradation**. **(a) **Scheme describing the generation of *S. cerevisiae *Swap strains by replacing the native *S. cerevisiae *promoter and 5' UTR with the orthologous sequences from *S. paradoxus*. Note that for each strain we modulate the regulatory region of only one gene while all other loci are identical to the wild-type (WT) *S. cerevisiae*. **(b) **Log_2_-ratio of mRNA levels in the swapped versus WT strains are shown for 34 genes with a predicted *cis*-dependent interspecies expression difference. Colors represent the concordance between the swapping effect and the inter-species expression difference. Expression changes of less than 40% were considered not significant, as the interspecies expression differences were approximately two-fold. **(c) **Log_2_-ratio of mRNA levels in the Swapped versus WT strains after transcriptional arrest. Colors indicate whether mRNA degradation rates are increased (red), decreased (green) or not significantly affected (black, *P *> 0.05). The average values from three technical replicates are shown along with regression lines. Error bars show standard deviation among three technical replicates, while both values are shown for experiments with two biological replicates (AAT2, MKC7, OXP1 and YML108W). **(d) **Same as (c) for a validation experiment of four selected genes with eight time points.

In previous work, we used interspecific hybrids to predict *cis*-dependent changes in gene expression for hundreds of genes, and a *cis*-dependent coupling of transcription and mRNA degradation. To validate these predictions and to examine whether the relevant *cis*-acting mutations may reside at the promoter or 5' UTRs, we generated *S. cerevisiae *strains in which upstream *cis*-regulatory sequences (200 to 700 bp of the sequence immediately upstream to the start codon of the gene of interest) have been replaced by the orthologous sequence from *S. paradoxus*, which are approximately 80% identical (Figure 1a; Table S1 in Additional file 1). For most genes examined (18 of 34), swapping affected mRNA levels and reproduced the previously predicted interspecies *cis*-dependent differences (Figure 1b).

To test whether the differences in mRNA levels reflect differences in transcription, mRNA degradation or both, we focused on nine of the genes examined above that were previously identified as having a *cis*-dependent coupling of transcription and mRNA degradation. We arrested transcription (with 1,10-phenanthroline) of strains with the wild type (WT) and with the swapped (Swapped) regulatory sequences of these genes, and measured the mRNA log_2_-ratio (Swapped/WT) at four time points (Figure 1c). The log_2_-ratio at the zero time point (before transcriptional arrest) reflects the difference in steady-state mRNA levels, while the change in that ratio along the time course reflects differences in mRNA degradation rates: if the swapped sequences increase mRNA degradation rates, then the mRNA log_2_-ratio (Swapped/WT) should decrease linearly across the time course (marked in red at Figure 1c), while if mRNA degradation decreases by the swapping then the mRNA log_2_-ratio should increase along the time course (marked in green). Strikingly, swapping affected both the steady-state mRNA levels and the rates of mRNA degradation for most genes examined (7 of 9), and in all of these cases the change in mRNA levels was opposite to that expected from the change in mRNA degradation: increased degradation was associated with increased (rather than decreased) steady state mRNA levels, and vice versa. This indicates that changes in mRNA degradation were accompanied by changes in transcription that had an opposite effect on mRNA levels, and that the changes in transcription were larger and thus determined the direction of change in mRNA levels. Four of these genes were chosen for validation (see Materials and methods), and their results were reproduced in additional experiments with eight time points (Figure 1d).

As a control, we examined the effect of the selection marker that is inserted upstream of the swapped *cis*-regulatory elements (Figure 1a). We found that inserting the markers at the same location, but without swapping the *cis*-regulatory sequences, had moderate effects on the expression levels of the regulated genes that did not account for most changes observed in the swapped strains (Figure S1a in Additional file 1). Assuming that the inserted marker and the swapped *cis*-regulatory region exert independent effects on the regulated genes, we subtracted the estimated marker effects from the observed effects of 17 swap strains described above. This analysis slightly increased the proportion of genes in which the swapped region reproduced the predicted *cis*-dependent interspecies differences (Figure S1a in Additional file 1). Surprisingly, for the two genes that were most sensitive to the selection marker (*Pex32 *and *Gal83*) the swapped strains also had an effect on mRNA degradation, suggesting that these effects could be due to the selection marker instead of the swapped regulatory region. Indeed, analysis of control strains (with the marker but without swapping the regulatory sequences) indicated that, for some genes, the selection marker affected both transcription and mRNA degradation (Figure S1b in Additional file 1). Thus, although the mechanism by which insertion of the selection marker affected the expression of these genes is unknown, this mechanism also coupled the transcription and mRNA degradation of some genes. A possible explanation is that these genes, which were chosen based on previous evidence of transcription-degradation coupling, are regulated by a mechanism that links their transcriptional activity to mRNA degradation and therefore that various means of modulating their transcriptional activity could induce an effect on mRNA degradation.

### Detailed analysis reveals coupled and uncoupled effects on transcription and mRNA degradation

**Figure 2:**
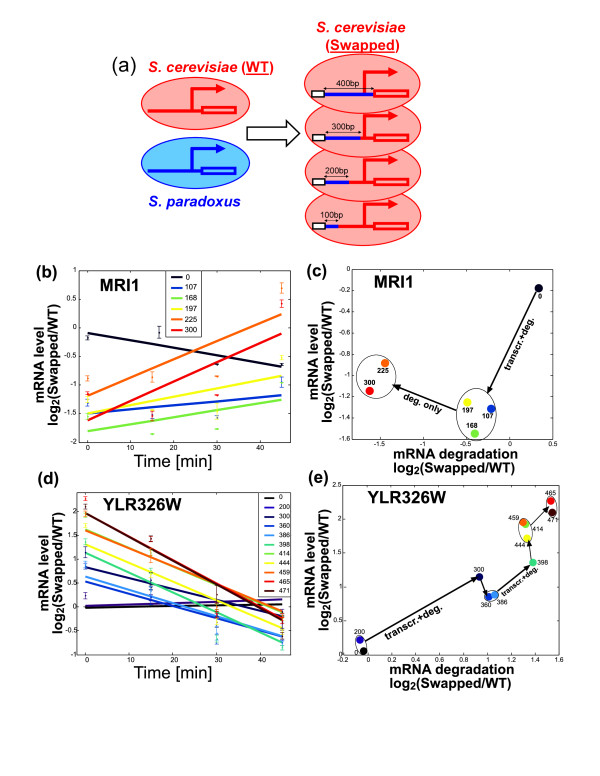
**Analysis of smaller swapped segments reveals multiple distinct effects on transcription, mRNA degradation and their coupling**. **(a) **Scheme describing the generation of a series of sequential *S. cerevisiae *Swap strains by replacing the different fractions of the native *S. cerevisiae *promoter and 5' UTR with the orthologous sequences from *S. paradoxus*. **(b,d) **Log_2_-ratio of mRNA levels in the Swapped versus WT strains after transcriptional arrest of MRI1 (b) and YLR326W (d). Error bars reflect standard error among two or four biological replicates (see Materials and methods). **(c,e) **Differences in mRNA degradation rates estimated using the linear least square fits shown in (b,d). Swap strains were classified into clusters by starting from a single cluster for all strains and separating consecutive strains into distinct clusters if they are significantly different (*P *< 0.05) in mRNA levels and/or mRNA degradation; the significant changes are noted by arrows.

Two genes were selected for a more detailed analysis (see Materials and methods). For each of the two genes, we generated a series of sequential *S. cerevisiae *strains where different fractions of the respective upstream regulatory sequence have been swapped by the orthologous region from *S. paradoxus *(Figure 2a). Each pair of 'adjacent' strains therefore differs by the species-of-origin (*S. cerevisiae *versus *S. paradoxus*) of a relatively small sequence element, and comparison of these strains uncovers the effect of mutations in this sequence only.

We first analyzed six swap strains of MRI1 and found that they are divided into two clusters that differ from the control strain (Figure 2b,c). The first cluster had a large decrease in mRNA levels (>2-fold) and a more subtle decrease in mRNA degradation. This cluster includes three strains with swapping of 107, 168 and 197 bp (out of the complete 300 bp swapped segment), indicating that the causal sequence is in the 107 bp upstream-most portion of the swapped segment. Consistent with previous studies [19,20], 5' RACE analysis (Figure S2 in Additional file 1) mapped the transcription start site of MRI1 to approximately position 250 within the swapped sequence (that is, 5' UTR of approximately 50 bp or even less) in the WT and Swapped strains, indicating that the causal sequence is a promoter element that couples a transcriptional effect with a (weaker) mRNA degradation effect.

In contrast, the second cluster showed an additional decrease of approximately two-fold in mRNA degradation and a more subtle increase in mRNA levels (compared to the first cluster). The increase in mRNA levels is expected from the decreased rates of mRNA degradation, suggesting that mRNA degradation is the primary effect and that transcription was either unaffected or only mildly affected by the additional swapped sequences. This cluster includes two strains with swapped segments of 225 and 300 bp, indicating that the causal sequences that distinguish this cluster from the first cluster are in the 197 to 225 bp region. This region is also upstream of the mapped transcription start site) and therefore reflects a promoter element that controls mRNA degradation.

Next we analyzed the swap strains of YLR326W (Figure 2d,e; Figure S3 in Additional file 1). Swapping of the entire 470 bp upstream region increased mRNA levels by approximately four-fold and mRNA degradation by approximately three-fold. The series of swap strains indicated a large effect of an upstream region (positions 200 to 300) and smaller effects of multiple other regions between positions 300 to 471 (Figure 2e). 5' RACE analysis (Figure S2 in Additional file 1) mapped the transcription start site to two positions corresponding approximately to positions 340 and 400, indicating that the upstream region with the large effect is a promoter element. This promoter element appears to increasemRNA degradation by approximately two-fold and transcription by approximately four-fold, thereby generating a net increase of two-fold in mRNA levels. The rest of the swapping effect, which was not accounted by this promoter element, was spread among eight distinct segments (positions 300 to 471) that collectively account for a 2-fold increase of mRNA level and a 1.5-fold increase of mRNA degradation. Each of these elements had effects that may be too weak to interpret individually (up to 30% changes in mRNA levels and degradation by each segment) as our measurements could have been affected by technical and biological variability. Nonetheless, we note that at least one of these segments (positions 386 to 398) appears to affect both transcription and mRNA degradation (each by approximately 30%) as these effect were statistically significant (*P *< 0.05; see Materials and methods) and were consistent with two independent experiments in which we swapped the containing segment 360 to 414 and observed a similar effect (Figure S3 in Additional file 1). This segment is found between the two distinct transcription start site positions of YLR326W and thus could control transcription and mRNA degradation either as a promoter or as a 5' UTR element.

### Gene-pairs that share a promoter have similar patterns of mRNA degradation

**Figure 3:**
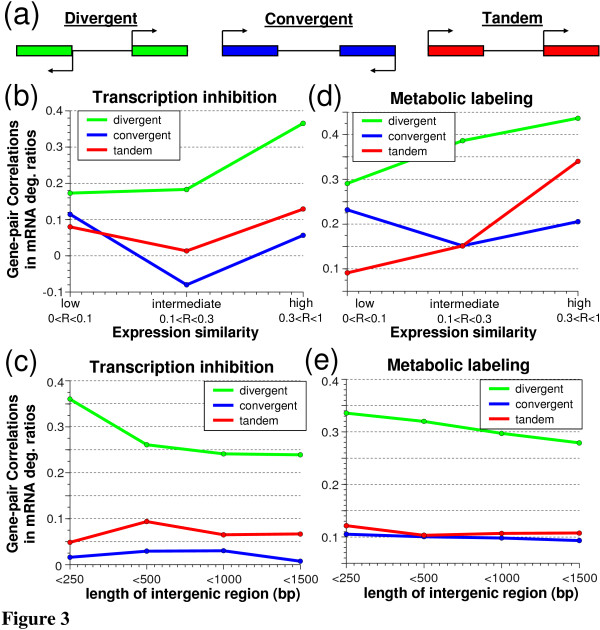
**Changes in mRNA degradation rates are correlated for genes that share an intergenic region**. **(a) **Schemes of the three neighboring gene-pair architectures. **(b-e) **Changes in mRNA degradation rates in response to stress were calculated using two data sets: one that used transcription inhibition [10] (b,c) and one that used metabolic labeling [9] (d,e). Consecutive gene pairs were binned according to their co-expression across a wide range of expression values [27] (b,d) or according to the length of the intergenic region (c,e). It is important to note that the correlation in expression, used to divide pairs according to their co-expression, is calculated over a very large set of samples (>1,700) resulting in expected low correlations even for genes that are co-expressed in some conditions. We chose the binning such that each bin contains roughly the same number of genes. Color indicates the gene-pair architecture (divergent, convergent and tandem). Spearman rank correlations are shown for each bin and each architecture demonstrating both the higher correlation for divergent pairs and the dependency on both the intergenic region length and the general tendency for co-expression.

To provide further statistical genome-wide support to the emerging notion that promoters might regulate mRNA degradation, we turned to examine the prediction that pairs of genes that are co-regulated by the same promoter elements will have similar patterns of mRNA degradation. Due to the compactness of the yeast genome the intergenic regions are often very short (typically lower than 1 kb) and hence neighboring gene pairs, with divergent orientation [21] (Figure 3a), might share promoter elements. Previous work has shown that divergent gene-pairs are somewhat correlated in expression in yeast [21] and in several other species [22]. We have thus asked whether these pairs will also show correlation in mRNA degradation. Interestingly, we found statistically significant, yet modest, correlations between mRNA degradation rates of divergent gene pairs, both with and without stress, and in the changes of degradation rates upon stress [10] (Pearson correlation = 0.24, 0.21, 0.25 and *P*-value = 2 × 10^-4^, 1 × 10^-3^, 1 × 10^-4^, respectively, in Figure S4 Additional file 1). We focus the following analysis in this section on the changes in mRNA degradation upon stress, as these reflect the condition-dependent regulation of mRNA degradation, while absolute rates of mRNA degradation may be more dependent on intrinsic features of the mRNA (for example, secondary structure) that are not linked to transcription. Nonetheless, similar analysis of absolute mRNA degradation rates (in both conditions) revealed similar patterns (Figures S5 and S6 in Additional file 1).

Sharing of upstream intergenic regions among divergent gene pairs might not indicate shared promoters, especially when the size of the intergenic region is relatively large [23]. Moreover, the correlated regulatory patterns of neighboring genes could have multiple other causes, including chromosomal proximity, which might cause adjacent genes to be co-regulated by various mechanisms, non-random arrangement of microarray probes [24,25] and the evolutionary tendency of functionally related (and thus often co-regulated) genes to be clustered in the genome [26]. However, these mechanisms should affect neighboring gene pairs of all orientations (Figure 3a), while promoter sharing should be more specific to divergent pairs. We have thus performed similar analysis for gene pairs of all three orientations to isolate the effect of promoter sharing from the additional factors that relate to genomic proximity (Figure 3b,c). Indeed, consistent with an effect of promoter sharing, gene-pairs with tandem and convergent architectures showed lower similarity in mRNA levels [21] and in mRNA degradation rates compared to divergent pairs. We further reasoned that promoter sharing is most likely in divergent gene-pairs that have short intergenic regions and high similarity in their mRNA expression profiles (and hence are likely co-regulated transcriptionally). We thus binned gene-pairs according to the length of their intergenic regions and their correlation in mRNA expression across a wide range of conditions [27] and examined the similarity in mRNA degradation profiles. As expected, the correlation in mRNA degradation rates between divergent gene pairs increased with decreasing intergenic length and with increasing expression similarity, and over all bins these correlations were more pronounced for divergent than for tandem or convergent gene pairs (Figure 3b,c).

We also examined the similarity in mRNA degradation as deduced from the more recent and completely different technology of metabolic labeling [9], in which transcription is measured directly along with mRNA levels, and mRNA decay is deduced. This methodology was used to evaluate the changes in transcription and in mRNA degradation in response to an environmental stress. Interestingly, also in this dataset we detected significant similarity between the changes in mRNA degradation rates of divergently transcribed gene pairs, and a lower similarity of convergent and tandem pairs (Figure 3d,e). In addition, the same dependency on the length of the intergenic region and transcriptional similarity is also observed for divergent genes in this dataset (Figure 3d,e). Notably, the extent of correlated mRNA degradation among divergent gene pairs was only slightly lower than the extent of correlated transcription profiles (Figure S7 in Additional file 1).

To add more support to the idea that shared promoter regulation results in coordination of post-transcriptional regulation of mRNA degradation, we examined pairs of genes that are not adjacent on the genome but share multiple promoter-associated regulators. For this we used physical measurements of protein-DNA interactions [28] and took all pairs of genes that share at least three regulators, and as a control we took pairs of genes that do not share a single regulator. We compared the differential effect of oxidative stress on mRNA degradation between the transcriptionally co-regulated gene pairs and the control gene pairs, and found that transcriptionally co-regulated gene pairs have significantly more similar patterns of mRNA degradation compared to the control gene pairs (Figure S8 in Additional file 1). Note that we controlled for the expression similarity [10] of the gene pairs and therefore this result is not due to the possibility that genes with similar expression patterns are more likely to have similar patterns of mRNA degradation. Instead, the increased similarity in mRNA degradation appears to be linked to the co-regulation by promoter-associated transcription factors.

In summary, although these analyses are correlative, they are based on patterns of hundreds of genes. Together they suggest that promoter sharing may contribute to the similarity in mRNA degradation profiles among neighboring genes, and provide support to the notion that promoters regulate mRNA degradation. Note that correlated mRNA degradation is observed not only for divergent but also, more weakly, for convergent and tandem gene pairs. This might indicate that some regulatory elements could regulate transcription and mRNA degradation from the terminator region, or alternatively that genomic proximity might enable sharing of transcription and degradation factors that are recruited by regulatory elements of one gene and then co-regulate the neighboring gene.

### Coupling of transcription and mRNA degradation in mammals

While the coordination of transcription and mRNA degradation has been studied primarily in yeast [6,7,11,12,17,18,29], previous studies also supported the possibility of a similar coordination in mammals. First, replacing the promoter of β-globin was shown to affect its mRNA degradation [30]. Second, deletion of a transcription regulator (Mat1 of the TFIIH complex) led to compensatory changes in transcription and mRNA degradation [31]. Third, genes with a 'peaked' response to environmental changes were shown to have concomitant changes in transcription and mRNA degradation [13,14]. To further examine this possibility we analyzed the changes in mammalian mRNA degradation rates between different cell types or species.

**Figure 4:**
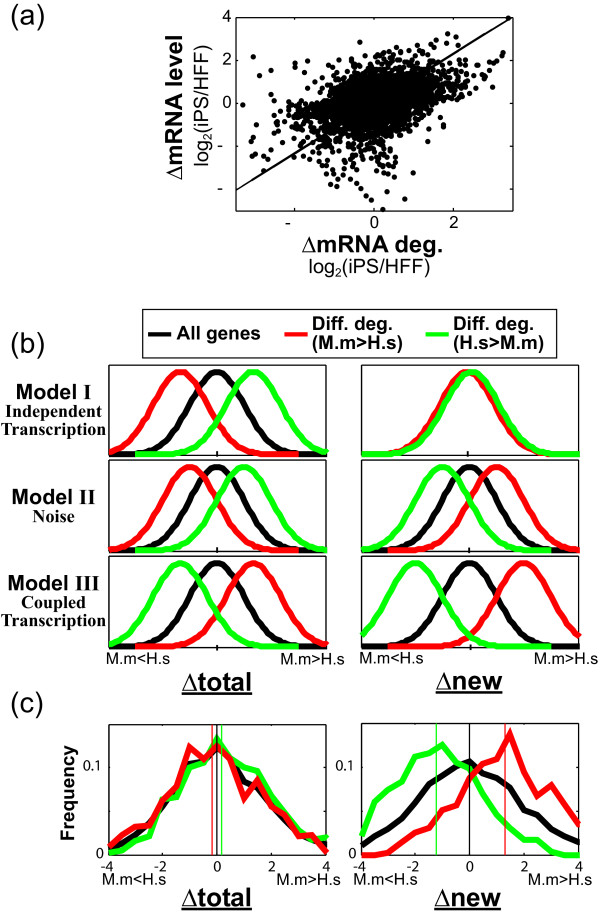
**Human-mouse comparison suggests conservation of the coupling between transcription and mRNA degradation**. **(a) **Scatter plot of cell type-dependent differences in mRNA levels (*y*-axis) and in mRNA degradation rates (*x*-axis) for 4,335 genes, quantified as log_2_(iPS/HFF). The Spearman correlation between the differences in mRNA degradation and mRNA levels is 0.44 and the corresponding linear least square fit is shown. **(b) **Theoretical distributions of *Δtotal *(left) and *Δnew *(right) according to three models, as detailed in the text, for all genes and for genes with differential mRNA degradation rates (red and green correspond to higher and lower degradation rates in mouse, respectively). **(c) **Observed distributions of *Δtotal *(left) and *Δnew *(right) following the color definitions in (b); vertical lines mark the distribution medians (*Δtotal*: -0.16, 0.19 and 0; *Δnew*: 1.33, 1.21 and 0; for red, green, and black, respectively). M.m and H.s denote *Mus musculus *and *Homo sapiens*.

We first examined the differences in mRNA degradation rates between induced pluripotent stem (iPS) cells and the human foreskin fibroblasts (HFFs) they were derived from, as recently measured by Neff *et al. *[32]. If changes in mRNA degradation upon induction of pluripotency are independent of the transcriptional changes in this process, then we would expect to see a negative correlation between changes in mRNA degradation rates and in mRNA levels, since increased mRNA degradation leads to decreased mRNA levels. However, consistent with the possibility that, as in yeast, changes in mRNA degradation are coupled to larger changes in transcription, we observed a significant positive correlation between changes in mRNA degradation rates and in mRNA levels, such that increased iPS cell mRNA degradation is preferentially associated with increased (rather than decreased) iPS cell mRNA levels (Figure 4a). This result reproduces similar observations in yeast [10,12] and suggests a global coupling between human transcription and mRNA degradation that is manifested in the induction of pluripotency.

We next performed two control analyses (Figure S9 in Additional file 1). First, in the above analysis the changes in mRNA levels and in mRNA degradation were calculated from the same experiment and this may introduce spurious correlations. To avoid potential technical correlations, we instead compared the mRNA degradation changes as defined by Neff *et al*. to changes in mRNA levels as defined by the average of four other (independent) studies that each compared pluripotent to fibroblast human cells [33]. Second, the rate of decrease in mRNA levels following transcriptional arrest is assumed to reflect only mRNA degradation but could also involve other effects, such as residual transcription or differential degradation along the time course, which would confound the estimation of mRNA degradation rates. While we cannot completely exclude such confounding effects, we reasoned that these are less likely to have an impact on genes in which the decrease in mRNA levels upon transcriptional arrest has the smallest deviation from the expected model of exponential decay. We thus restricted the control analysis to 12% of the genes with the best fit to the expected mRNA degradation model. In both controls we observed a strong positive correlation between changes in mRNA degradation and in mRNA levels (R > 0.3, *P *< 0.001; Figure S9 in Additional file 1).

Next, following our previous work in yeast [12], we sought to examine the possibility of transcription-degradation coupling from an evolutionary perspective through interspecies comparison of transcription and mRNA degradation. To this end, we compared mRNA degradation rates between human B cells (BL41) and murine NIH-3T3 fibroblasts [34]. The two datasets were produced by the same lab with the same methodology, but for non-orthologous cell types and therefore the differences reflect both evolutionary and developmental changes in gene regulation. This analysis is complementary to the above analysis of HFFs versus iPS cells as it involves a different comparison of cell lines, but most importantly as it is based on a completely different methodology of metabolic labeling of newly transcribed mRNA with 4-thiouridine for 1 hour. Thus, transcription rates were estimated by the amount of newly transcribed mRNA, and degradation rates were estimated by comparing the newly transcribed to total mRNA levels (a high relative fraction of newly transcribed mRNAs indicates rapid turnover).

We calculated interspecies differences in total mRNA levels (*Δtotal*) and in newly transcribed mRNA (*Δnew*), both reflecting log_2_-ratios of mouse versus human. *Δtotal *and *Δnew *were highly correlated (R = 0.88; Figure S10 in Additional file 1), indicating that most interspecies differences in total mRNA levels are due to transcriptional differences, or to differences in microarray probe efficiencies for orthologous human and mouse genes. Nonetheless, many genes had significant differences between *Δtotal *and *Δnew*, indicating differential mRNA degradation rates (for example, if *Δtotal *>*Δnew*, then the change in total mRNA levels cannot be accounted for by changes in transcription and suggests a change in mRNA degradation). To understand the connection between changes in mRNA degradation and changes in transcription, we considered three models, and the expected distributions of *Δtotal *and *Δnew *according to these models are shown for genes with increased (red) or decreased (green) mRNA degradation, in mouse compared to human (Figure 4b). Briefly, if differences in transcription are independent of the differences in mRNA degradation (Model I), then we expect no effect (equal distribution for the two gene sets) in transcription (*Δnew*), but a significant effect of mRNA degradation on total mRNA levels (*Δtotal*). If differences in mRNA degradation primarily reflect measurement errors in *Δnew *and *Δtotal *(Model II), then we expect moderate shifts in the distributions of both *Δnew *and *Δtotal *(for example, increased mRNA degradation, *Δnew *- *Δtotal > 0*, would be identified preferentially at genes with *Δnew *>0 and *Δtotal*<0), as observed in comparison of unrelated (non-orthologous) genes (Figure S11 in Additional file 1). Finally, if, as in yeast [10,12], differences in mRNA degradation are typically coupled to larger differences in transcription (Model III), then we expect both *Δnew *and *Δtotal *to be shifted such that increased degradation is associated with increased newly transcribed and total mRNAs.

Interestingly, comparison of human-mouse orthologs shows that the distributions of *Δtotal *are, in fact, quite similar for genes with increased and decreased mRNA degradation, and are only slightly shifted (Figure 4c). As both of the first two models (Independent transcription or Noise) predict a large shift of *Δtotal *in the same direction, the small shift in *Δtotal *suggests compensation by transcriptional effects according to the third model (Coupled transcription), which predicts an opposite shift. Consistent with this possibility, we observe a remarkable shift in the distribution of *Δnew*. The shift in *Δnew *is much larger than the shift in *Δtotal*, which can only be explained by the third model. These results indicate that, on average, differences in mRNA degradation are compensated for by transcriptional effects and may be explained by a combination of the above models - in some genes transcriptional effects are independent of (and do not compensate) the changes in mRNA degradation, while in other genes transcriptional effects over-compensate for the changes in mRNA degradation and generate an opposite change of total mRNA levels.

## Discussion

We previously predicted (using interspecific hybrids) *cis*-dependent evolutionary changes in mRNA levels and in mRNA degradation of hundreds of yeast genes. In the first part of this work we have validated these predictions and demonstrated that the relevant mutations often reside at the swapped upstream regulatory regions containing promoters and 5' UTRs. We further analyzed the contribution of different upstream segments to the divergence of two genes and identified promoter and UTR elements that affect transcription and mRNA degradation. Promoter alterations could influence the recruitment of RNA-binding factors, such as Rpb4/7, that would shuttle with the mRNA to the cytoplasm and affect their degradation and/or translation. Other evidence for this model comes from our observation that gene pairs that share a promoter tend to have similar patterns of mRNA degradation across the yeast genome. This suggests that promoter-mediated effects on mRNA degradation often act in both directions of a bidirectional promoter.

Recent studies identified two mechanisms by which promoter elements affect mRNA degradation [17,18] but, as noted above, these mechanisms are unlikely to account for our results. This may indicate the existence of multiple independent mechanisms for coupling transcription to mRNA degradation. Alternatively, it is possible that these seemingly distinct cases in fact reflect a common general coupling mechanism whose activity is modulated by multiple different regulators. Accordingly, general coupling factors (for example, Rpb4/7 and/or Ccr4-Not) may be recruited to a large fraction of the transcribed genes by yet unknown mechanisms and their gene-specific activity could then be fine-tuned by various transcription regulators, including Rap1 and cell-cycle regulators. This model is consistent with the various observations of a global (rather than gene-specific) coordination of transcription and mRNA degradation, as shown by this and previous studies [6,11,12]. This model is further supported by observations that the same gene often displays a coupling of transcription and mRNA degradation through multiple distinct mechanisms. First, Bregman *et al. *[18] noted that apart from the large effect of Rap1 binding sites, two other promoter elements (P_ACT1_, P_RPL30_) also had independent effects on the mRNA degradation rate of their construct. Second, our analysis of YLR326W identified distinct *cis*-regulatory elements that affected both transcription and mRNA degradation. Third, we found that genes in which transcription and mRNA degradation were previously shown to be coupled by *cis*-mutations were also coupled through the insertion of a selection marker. It would therefore be interesting to examine if Rpb4/7 and Ccr4-Not interact differently with mRNAs of coupled versus uncoupled genes.

Finally, we analyzed differences in mRNA degradation between mammalian cell-types or species and provide evidence for a global coordination between transcription and mRNA degradation. Importantly, the analyses of mammalian cells, as well as the analyses of divergent yeast gene pairs, were both performed on two different datasets of mRNA degradation rates, with one that used transcriptional arrest and another that used metabolic labeling. These completely different technologies produce distinct estimates of mRNA degradation rates, yet in our analyses they show consistent results, further supporting our conclusions. Our observation of similar transcription-degradation coupling in yeast and in mammals may suggest that coupling is an evolutionarily conserved phenomenon. Given the conservation of the protein complexes Rpb4/7 and Ccr4-Not, it is tempting to speculate that similar mechanisms govern this coupling in yeast and in mammals and therefore that a promoter-mediated coupling exists also in mammals. Consistent with this possibility, recent work in a human embryonic cell line identified several mRNA-binding transcription regulators, as well as an overall enrichment of interactions between mRNA-binding proteins and transcriptional regulators, demonstrating the many potential links between transcription and post-transcriptional regulation [35]. Alternatively, it is possible that the observed coupling evolved independently in yeast and in mammals and therefore reflects convergent evolution.

## Conclusions

Our results demonstrate, at the genomic scale, that transcription and mRNA degradation are coordinated in yeast as well as in humans. In yeast, detailed experimental and computational analysis further indicates that this coupling is driven by promoters and cannot be accounted for by two recently described mechanisms, suggesting that multiple mechanisms influence the coupling of transcription and mRNA degradation. Additional work would be needed to decipher the relevant mechanisms and their implications.

## Materials and methods

### Selection of genes for swapping experiments

We first selected 40 genes for swapping experiments based on a high degree of inter-species *cis*-dependent differences in mRNA levels and consistency of these *cis*-effects among several microarray experiments [36]. The first half of the genes (20 genes) was selected among the genes with a previous observation of transcription-degradation coupling [12] and was selected to have equal representation of OPN and DPN promoters [37]. Of these 40 genes, six were discarded due to technical problems in generating the swap strains, and for the rest we examined the effect of swapping on mRNA levels. Of these, 10 genes with a previous observation of transcription-degradation coupling were selected at random and for these we examined the effect of swapping on mRNA degradation. One gene was excluded due to lack of reproducibility, and for the rest we estimated the swapping effect. Of these, the four genes in which the swapping effects on mRNA degradation appeared most reliable were chosen for further validation with a longer time course of eight time points. The swapping effect of one of these genes (GAL83) was largely reproduced in a control strain in which we only inserted the selection marker, while the swapping effect of another gene (CEX1) was relatively weak, and thus we further examined in detail (with a series of swap strains) the two remaining genes, MRI1 and YLR326W. The selection of swapped segments was based on the ability to design efficient primers, the location of neighboring genes and the location of known *cis*-regulatory motifs.

### Generating swap strains

Wild-type *S. cerevisiae *BY4741 was compared to BY4741 strains in which we replaced the promoter and 5' UTR segment of an individual gene with the orthologous segment from *S. paradoxus *CBS432 (Table S1 in Additional file 1). The orthologous segments were first cloned and inserted into a pBS7 plasmid next to a kanamycin selection marker, and the resulting construct (selection marker upstream of a regulatory segment) was inserted into *S. cerevisiae*.

In the first set of 17 genes, including all 9 genes for which we examined mRNA degradation, the *S. cerevisiae *upstream regions were replaced by this construct. For these genes we also generated control strains in which only the kanamycin marker was inserted (at the same position, but without replacing the regulatory region) and for two of these genes (YLR326W and MRI1) we generated a series of strains where different portions of the upstream regulatory regions were replaced, thus generating chimeric intergenic regions composed of partial *S. paradoxus *and partial *S. cerevisiae *segments (together encompassing the original length of the upstream region) and an upstream selection marker.

A caveat of the above swapping approach is that the swapped region might contain an element (promoter or terminator) that regulates the adjacent upstream gene (not the one being studied), and these elements are not only swapped but are also being separated from the adjacent gene by the selection marker. To avoid this potential effect, in the second set of 17 genes we did not replace the *S. cerevisiae *regulatory region but instead inserted the construct downstream of the *S. cerevisiae *regulatory region (directly upstream of the start codon), thereby maintaining the *S. cerevisiae *regulatory region next to the adjacent gene but also inserting the *S. paradoxus *regulatory region next to the gene being studied. For these genes we did not generate control strains. For measurements of mRNA degradation rates, the swap and control *S. cerevisiae *strains were mated with a wild-type *S. paradoxus *(CBS432) in order to allow comparison of the *S. cerevisiae *allele (whose regulatory sequences are swapped) with the orthologous *S. paradoxus *allele, and also because transcriptional arrest experiments appeared to be more efficient in diploid strains (not shown).

### Comparison of wild-type and swap strains

Real time PCR was performed for the WT and swap strains with duplicates or triplicates. We compared the mRNA levels of the genes whose regulatory sequences were swapped to reference genes that are not expected to be affected by the swapping and thus provide a normalization factor for comparison between the WT and the swap strains. The effect of swapping was thus quantified as:

$$\Delta \text{swap}={\text{log}}_{2}\left[\left(\text{S}{\text{W}}_{\text{g}}\text{/W}{\text{T}}_{\text{g}}\right)/\left(\text{S}{\text{W}}_{\text{c}}\text{/W}{\text{T}}_{\text{c}}\right)\right]$$

where SW and WT denote the swapped and wild-type strains, respectively, and g and c denote the examined gene and its reference, respectively. ACT1 was used as a reference gene for PCR normalization in the analysis of mRNA levels (Figure 1b). Preliminary analysis suggested that ACT1 may be affected by the phenanthroline treatment and thus we did not use it as a reference in any of the mRNA degradation experiments. In the initial mRNA degradation experiments (Figure 1c; Figure S1b in Additional file 1) we used ARO80 as a reference gene. We note that our experiments are designed to measure changes in mRNA degradation due to swapping of a single *cis*-regulatory segment (rather than absolute mRNA degradation rates) and hence are less dependent on the choice of reference gene as long as the reference gene is not directly affected by the swapped regulatory segment. Nonetheless, for the validation experiments (Figure 1d) we sought to use different reference genes in order to demonstrate the reproducibility of the observations and their lack of dependence on the reference gene. For this reason and also in order to reduce the overall number of experiments, we used a circular design whereby each of the four genes being validated also served as a reference gene for one of the other genes: each gene was measured in the WT and in two swap strains including the swap of that gene and of another gene, where it served as a reference (GAL83 - YLR326W and CEX1 - MRI1 controlled for one another).

### Analysis of differential mRNA degradation

Transcription was arrested by addition of 150 μM 1,10-phenanthroline to the WT and swap hybrid strains and monitored mRNA levels at four or eight time points. Least squares linear regression was used to estimate the slope along the time-course, which reflects the effect of swapping on rates of mRNA degradation. For example, a slope of -0.05 indicates that after 20 minutes the relative mRNA level of the swap strain decreases by 2-fold (log_2_[SW_g_/WT_g_] = -0.05 × 20 = 0.5). These slopes were also used to estimate the relative effect of the swapping on mRNA degradation rates (shown in Figure 2) by comparison with the average half-life (t_1/2_) of the native *S. cerevisiae *gene from two previous studies [10,38]:

$$\Delta \text{deg}={\text{log}}_{2}\left[\left(1\text{/}{\text{t}}_{1/2}-\text{slope}\right)\text{/}\left(1\text{/}{\text{t}}_{1/2}\right)\right].$$

To determine significance of the differences in mRNA degradation rates between WT and Swapped (Figure 1), or between swapping of different lengths (Figure 2), we performed paired *t*-tests, evaluating the differences between the changes in mRNA levels at 20 minute intervals. In each experiment we can quantify the mRNA degradation rate at three 20 minute intervals:

$$\Delta_{\text{i},\text{i}+20}={\text{log}}_{2}\left({\text{m}}_{\text{i + 20}}/{\text{m}}_{\text{i}}\right)$$

where m_i _is the relative mRNA level at time i compared to the reference gene, for i = 0,20,40. The *t*-test is performed over the differences between paired effects from the two strains (for example, Swapped and WT): Δ_i,i+20_(SW,k) - Δ_i,i+20_(WT,k), for i = 0,20,40 and k = 1..n, where n is the number of biological repeats. The pairing of corresponding time intervals is important because the pattern of changes along the time course is not exactly linear and is often gene-specific. By comparing the same gene between different strains and restricting the comparison to corresponding intervals we can largely eliminate the variability along the time course and focus on the variability between strains (that is, the swapping effect).

mRNA degradation experiments were typically performed with three technical replicates and with two biological replicates, with three exceptions: (1) YLR326W swaps of 200, 386, 398, 414, 444, 459 and 471 bp (Figure 2) were done in four replicates; (2) complete swaps of YLR326W, CEX1, GAL83 and MRI1 were analyzed without biological replicates but in two different experiments - an initial experiment with four time-points (Figure 1c) and a later experiment with eight time points (Figure 1d); (3) the complete swap of PEX32 was analyzed without biological replicates due to the strong effect of the selection marker.

### Human-mouse comparison

Total and newly transcribed mRNA levels for human BL41 and murine NIH-3T3 cell lines with three replicates were taken from Gene Expression Omnibus (GSE10026). We averaged the three replicates, and compared the averages of selected probes for orthologous genes (following the orthologs and probe selection of Friedel *et al. *[34]). We calculated log_2_(mouse/human) for total (*Δtotal*) and nascent (*Δnew*) mRNA levels and centered these log_2_-ratios on zero. Human and mouse half-lives were taken from the supplement of Friedel *et al*. and genes with differential mRNA degradation rates were identified as those with at least 1.75-fold difference between the species both according to the Friedel *et al*. half-life estimates and according to (*Δnew *- *Δtotal*). We filtered out genes with very low hybridization intensities and/or very large interspecies differences, although the trends shown in Figure 4 were maintained without filtering or with different stringencies of filtering (not shown). This analysis included 4,209 genes (shown in black in Figure 4), with 729 genes defined as higher mRNA degradation rates in mouse (red) and 629 genes defined as higher mRNA degradation rates in human (green).

### 5' RACE

RNA was extracted from WT and two mutant strains using Yeast MasterPure kit (Epicenter Biotechnologies, **Madison, WI, USA**), including DNAse treatment to reduce contaminations of genomic DNA. To discriminate between mature capped mRNA and mRNA degradation products, RNA samples were dephosphorylated using FastAP thermosensitive Alkaline Phosphatase (Thermo Scientific, Waltham, MA, USA). RNA was then cleaned using RNeasy MinElute Cleanup kit (Qiagen, Venlo, Netherlands). 5' Caps were then removed using Tobacco acid pyrophosphatase (TAP) enzyme (Epicenter Biotechnologies) to generate 5' monophosphate for mature intact mRNAs and RNA was cleaned using RNeasy kit. A unique RNA adaptor (UCUUUCCCUACACGACGCUCUUCCGAUCUGCGC) was ligated using T4 RNA ligase (New England BioLabs, Ipswich, Ma, USA) with incubation of 1.5 hours at 22°C, and RNA was cleaned using RNeasy kit. First strand cDNA was generated from total amount of RNA ligation product, random hexamer primers and M-MLV reverse transcriptase. cDNA (2 μl) was used as template for each following PCR reaction. To estimate the length of the 5' UTR a reverse primer located downstream of the start codon and a forward primer on the adaptor (ATGATACGGCGACCACCGAGATCTACACTCTTTCCCTACACGACGCTCTT) were used together and products were run on a 1.5% agarose gel. Reverse primer sequences: CGGTAGAAGTGCAAGTAATGG for YLR326W and for ATAAATTAACGGCTGTGGGTC. Primers were validated using an additional forward primer located on the ATG of each gene, ATGTCAGGGTTCATTAAGAGC for YLR326W and ATGTCGTTGGAAGCCATCGTC for YPR118W.

## Abbreviations

5' RACE: rapid amplification of 5' complementary DNA ends; HFF: human foreskin fibroblast; iPS: induced pluripotent stem cells; PCR: polymerase chain reaction; UTR: untranslated region; WT: wild type.

## Authors' contributions

MDB, OS, YP and IT conceived and designed the experiments. MDB and OS performed the experiments. IT, YP, OS and YM analyzed data. IT, YP and OS wrote the manuscript. All authors read and approved the final manuscript.

## Supplementary Material

Additional file 1 **Table S1 and Figures S1 to 11**. Table S1: genes examined by swapping of regulatory sequences. Figure S1: the effects of inserted selection markers on transcription and mRNA degradation. Figure S2: 5' RACE analysis. Figure S3: independent experiments reproduce the YLR326W expression differences between strains with swap segments of different lengths. Figure S4: correlation in different mRNA degradation measures between consecutive gene pairs with different genomic architectures. Figure S5: correlation of basal mRNA degradation rates for consecutive gene pairs. Figure S6: correlation of stress mRNA degradation rates for consecutive gene pairs. Figure S7: correlation of nascent transcription rates for consecutive gene pairs. Figure S8: similarity in mRNA degradation changes due to oxidative stress as a function of similarity in mRNA level changes in oxidative stress, for pairs of genes that share or do not share multiple promoter-associated regulators. Figure S9: the positive correlation between cell type-dependent differences (iPS cells versus HFFs) in mRNA levels and mRNA degradation is maintained in two control analyses. Figure S10: scatter plot of interspecies differences in total mRNA levels and in nascent mRNA levels. Figure S11: human-mouse comparison of non-orthologous genes reproduces the expected behavior of the Noise model (Model II in Figure 4)[39]. Click here for file
