# Supplementary material for: Widespread promoter-mediated coordination of transcription and mRNA degradation
Source: Genome Biol. 2012 Dec 13;13(12):R114. doi: 10.1186/gb-2012-13-12-r114 (PMC4056365; doi:10.1186/gb-2012-13-12-r114)
Supplement: Additional file 1 — Table S1 and Figures S1 to 11. Table S1: genes examined by swapping of regulatory sequences. Figure S1: the effects of inserted selection markers on transcription and mRNA degradation. Figure S2: 5' RACE analysis. Figure S3: independent experiments reproduce the YLR326W expression differences between strains with swap segments of different lengths. Figure S4: correlation in different mRNA degradation measures between consecutive gene pairs with different genomic architectures. Figure S5: correlation of basal mRNA degradation rates for consecutive gene pairs. Figure S6: correlation of stress mRNA degradation rates for consecutive gene pairs. Figure S7: correlation of nascent transcription rates for consecutive gene pairs. Figure S8: similarity in mRNA degradation changes due to oxidative stress as a function of similarity in mRNA level changes in oxidative stress, for pairs of genes that share or do not share multiple promoter-associated regulators. Figure S9: the positive correlation between cell type-dependent differences (iPS cells versus HFFs) in mRNA levels and mRNA degradation is maintained in two control analyses. Figure S10: scatter plot of interspecies differences in total mRNA levels and in nascent mRNA levels. Figure S11: human-mouse comparison of non-orthologous genes reproduces the expected behavior of the Noise model (Model II in Figure 4)[39]. [file gb-2012-13-12-r114-S1.PDF]

Supplementary information

Table S1. Genes examined by swapping of regulatory sequences

Gene	Swapped (bp)	*Cis*-effect[a]	Swap effect[b]	Marker effect[c]
YLR326W	470	1.62	1.9	0.1
NUP84	282	1.6	0.82	0.25
IRC19	253	1.59	0.9	
YML108W	246	1.51	0.7	0.85
ACM1	309	1.46	0.72	
TDA2	262	1.46	3.15	0.38
URA10	588	1.4	0.83	
YDL183C	609	1.34	-0.32	
CBS1	388	1.19	0.94	
RCR1	222	1.13	1.77	
DDI1	258	1.13	-0.37	
DAL2	417	1.12	3.0	
ERG26	231	1.08	1.2	
ATG12	215	1.07	1.39	
LTP1	404	0.85	-0.17	-0.68
GAL83	295	0.66	1.6	1.85
CEX1	300	0.64	0.27	-0.76
EPS1	402	-0.67	1.03	
MKC7	370	-0.69	-0.18	-0.3
AAT2	394	-0.72	-0.43	-0.39
RDS1	530	-0.89	-0.97	0.76
YDR266C	351	-0.9	0.28	0.004
OXP1	297	-1.1	-0.8	-0.28
URA4	585	-1.11	0.23	
NIS1	600	-1.22	0.34	
ANT1	457	-1.23	-0.043	
PEX32	273	-1.23	1.71	2.74
MRI1	301	-1.25	-0.91	0.23
YUH1	410	-1.26	0.27	
ECM32	705	-1.36	-1.16	0.78
PCL7	601	-1.4	-0.58	
SOL3	245	-1.41	1.68	0.85
SAD1	259	-1.45	-0.061	
NAS6	461	-1.67	0.01	
YBR197C	179	-1.78	1.57	0.77

[a] hybrid mRNA log_2-ratio (*S. paradoxus* allele divided by *S. cerevisiae* allele), averaged over several microarray experiments at different conditions [36] and upon deletion of different genes [39].

[b] mRNA log_2-ratio (Swapped divided by WT)

[c] hybrid log_2-ratio (control divided by WT)

Figure S1. The effects of inserted selection markers on transcription and mRNA degradation.

(a) Selection markers partially accounts for the effects of the swapped strains on steady state mRNA level. Log_2-ratio of mRNA levels in the control vs. WT strains are shown for 17 of the 34 strains examined in Fig. 1 (genes are shown in the same order as in Fig. 1). The markers had significant effects (>40%) for 10 of the 17 genes. To estimate the swapping effect we compared the effect of the swapping with marker (as shown in Fig. 1) to the effect of the marker only (shown here) and assumed that the marker and the swapping effects are additive. The inferred swapping effects were significant for 12 of the 17 genes, with ten genes in the expected direction (as observed for the inter-species comparison) and two genes in the opposite direction. Inclusion of the marker control therefore increased the fraction of genes where the swapping effect correctly reproduces the interspecies *cis*-differences from 9/17 (Fig. 1) to 10/17 (here). Colors represent the inferred swapping and marker effects: three genes did not have a significant marker or swap effects (shown in white); four genes had only a significant swap effects in the expected direction (shown in red); two genes had only a significant marker effect (shown in blue); eight genes had both a significant swapping and a significant marker effects, and of these the swapping effect was in the expected direction for six genes (shown in green) and in the opposite direction for 2 genes (shown in black).

(b) Selection markers affect transcription and mRNA degradation. Log_2-ratio of mRNA levels in the control vs. WT strains after transcriptional arrest. Colors indicate whether mRNA degradation rates are increased (red), or not significantly affected (black, $P>0.05$). Error-bars reflect standard error among three technical replicates. Note also that the zero time point is consistent with the independent experiments reported in the 'Marker effect' column of Table S1.

Figure S2: 5' RACE analysis. Results of 5' RACE were run on gel to estimate the length of the 5' UTR and compare mutant and WT strains. For both genes, WT and mutant strains show similar patterns of TSS. Note that two distinct TSSs can be seen for YLR326W gene. The lengths of both YLR326W bands are smaller than 500 bp, and the length of the MRI1 band is smaller than 450 bp. Given the length of the adaptors and the positions of the ORF-internal primers (see scheme at the left) this indicates that the length of the 5'-UTR is smaller than 154 bp for YLR326W and smaller than 55 bp for MRI1. The swapped segments with the main transcription-degradation coupling effects were 200-300 bp (YLR326W) and 0-107 bp (MRI1) within the complete swapped segments (see Fig. 2), which corresponds to 190, 194 bp upstream of the ATGs of these two genes, respectively. Thus, in both cases these swapped segments were clearly upstream of the TSS and reflect promoter elements that couple transcription and mRNA degradation.

Figure S3. Independent experiments reproduce the YLR326W expression differences between strains with swap segments of different lengths. Error-bars reflect standard error among two biological replicates.

Figure S4. Correlation in different mRNA degradation measures between consecutive gene pairs with different genomic architectures. Each plot shows the correlation between all gene-pairs separated by less than 800bp with a certain arrangement (divergent, convergent and tandem architectures, from left to right, as noted at the top of the figure) and based on a certain dataset of mRNA degradation (basel, stress and ratio, from top to bottom, as noted below each plot). Within each

plot, points represent gene-pairs. The Spearman correlation values and associated p-values are indicated within each plot.

Figure S5. Correlation of basal mRNA degradation rates for consecutive gene pairs. Similar to figure 3 only using basal mRNA degradation rates instead of the ratio between stress and basal.

Figure S6. Correlation of stress mRNA degradation rates for consecutive gene pairs. Similar to figure 3 only using mRNA degradation rates measured in stress instead of the ratio between stress and basal.

Figure S7. Correlation of nascent transcription rates for consecutive gene pairs. Similar analysis to figure 3 using measurements of nascent transcription rate [9]. Top and bottom panels show binning by co-expression or intergenic length respectively. Columns refer to changes in transcription rates, basal, and stress transcription rates, from left to right.

Figure S8. Similarity in mRNA degradation changes due to oxidative stress as a function of similarity in mRNA level changes in oxidative stress, for pairs of genes that share or do not share multiple promoter-associated regulators. The difference in mRNA degradation fold change (Y axis) is plotted as a function of mRNA level correlation in oxidative stress (X axis) for pairs of genes that share at least 3 promoter regulators or none (green and black points respectively). Thick lines represent a moving window average of 250 points showing a significantly stronger similarity for genes that share promoter regulators compared to genes that do not share, despite having the same level of correlation in mRNA abundance. The difference between gene-pairs with or without shared regulators is significant ($P<10^{-16}$) based on a two-sampled t-test comparing all gene-pairs with an expression level correlation larger than 0.6.

Figure S9. The positive correlation between cell type-dependent differences (iPS versus HFF) in mRNA levels and mRNA degradation is maintained in two control analyses. (a) Differences in mRNA degradation from Neff et al. [32] were compared to differences in mRNA levels which were averaged over four other studies that compared gene expression between pluripotent and fibroblast human cells [33]. Genes were included in the analysis only if they were defined as having differential expression (pluripotent versus fibroblast) in at least three of the four studies and their expression log-ratios were averaged over these studies. The Spearman correlation between the differences in mRNA degradation and mRNA levels is 0.31 and the corresponding linear least square fit is shown. (b) Differences in mRNA degradation and mRNA levels (from Neff et al.) were compared only among genes in which the fit of the observed data (decrease in mRNA levels after transcriptional arrest) to the exponential decay model had a p-value smaller than 0.001, which retained only 12% of the genes analyzed with the original cutoff of 0.05 [32]. The Spearman correlation between the differences in mRNA degradation and mRNA levels is 0.55 and the corresponding linear least square fit is shown.

Figure S10. Scatter plot of interspecies differences, quantified as $\log_2$(mouse/human), in total mRNA levels (*Δtotal*, *x*-axis) and in nascent mRNA levels (*Δnascent*, *y*-axis). Genes with differential mRNA degradation rates in the two species are marked in red (higher degradation rates in mouse) and in green (higher degradation rates in human).

Figure S11. Human-mouse comparison of non-orthologous genes reproduces the expected behavior of the Noise model (Model II in Fig. 4). 200,000 pairs of human and mouse genes were sampled at random, and were processed in the same way as the orthologous genes in Fig. 4. For each pair of genes we calculated *Δtotal*, *Δnascent* and differential half-life, centered these three distributions on zero, and identified gene-pairs with differential mRNA degradation rates, following the definitions in the orthologs analysis. The distributions of *Δtotal* and *Δnascent* are shown for all gene-pairs (black) and for those with higher mRNA degradation rates in mouse (red) and in human (green).

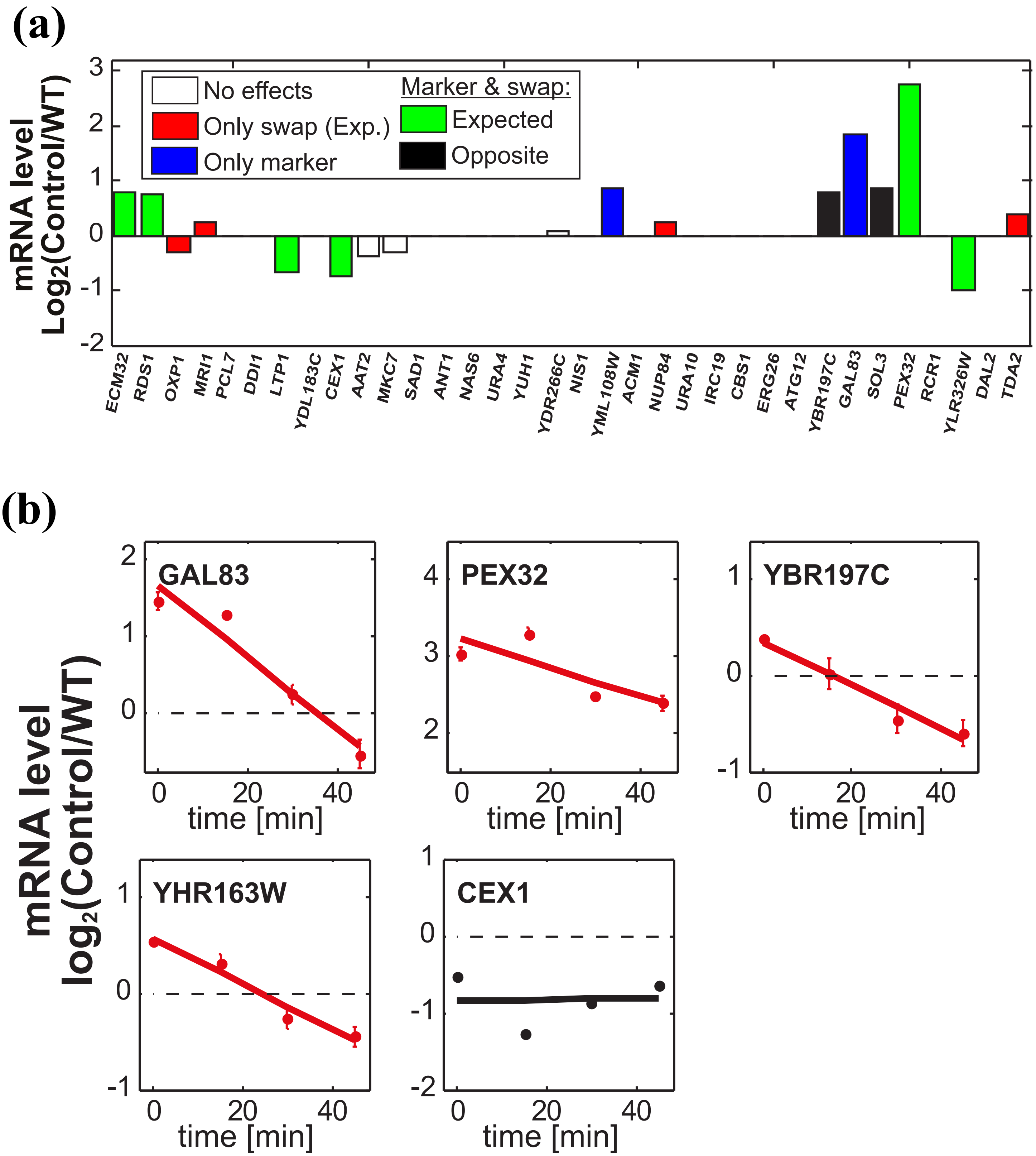

(a)
mRNA level Log2(Control/WT)
No effects
Only swap (Exp.)
Only marker
Marker & swap:
Expected
Opposite
ECM32 RDS1 OXP1 MRI1 PCL7 DDI1 LTP1 YDL183C CEX1 AAT2 MKC7 SAD1 ANT1 NAS6 URA4 YUH1 YDR266C NIS1 YML108W ACM1 NUP84 URA10 IRC19 CBS1 ERG26 ATG12 YBR197C GAL83 SOL3 PEX32 RCR1 YLR326W DAL2 TDA2
(b)
mRNA level log2(Control/WT)
GAL83
PEX32
YBR197C
YHR163W
CEX1
time [min]

Figure S1

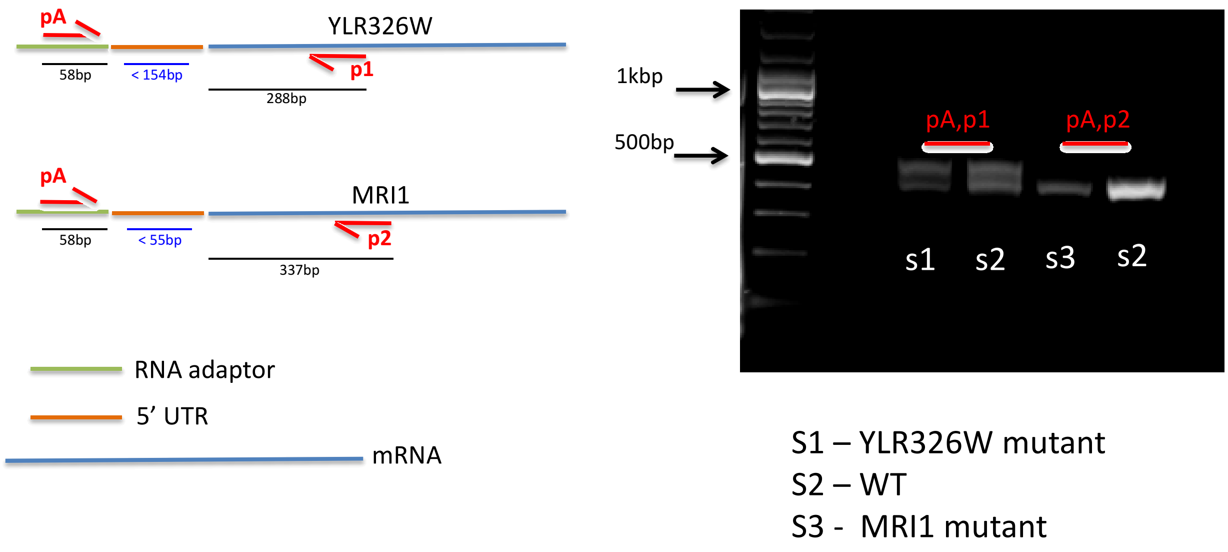

Figure S2

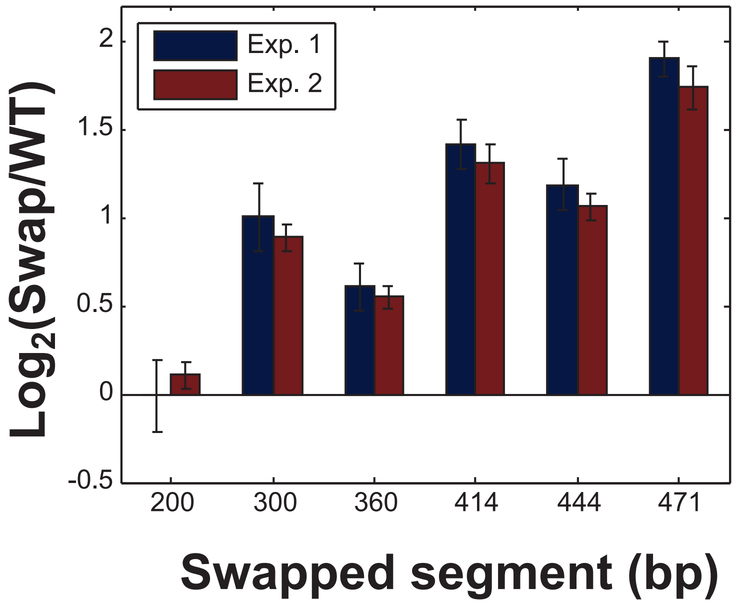

Figure S3

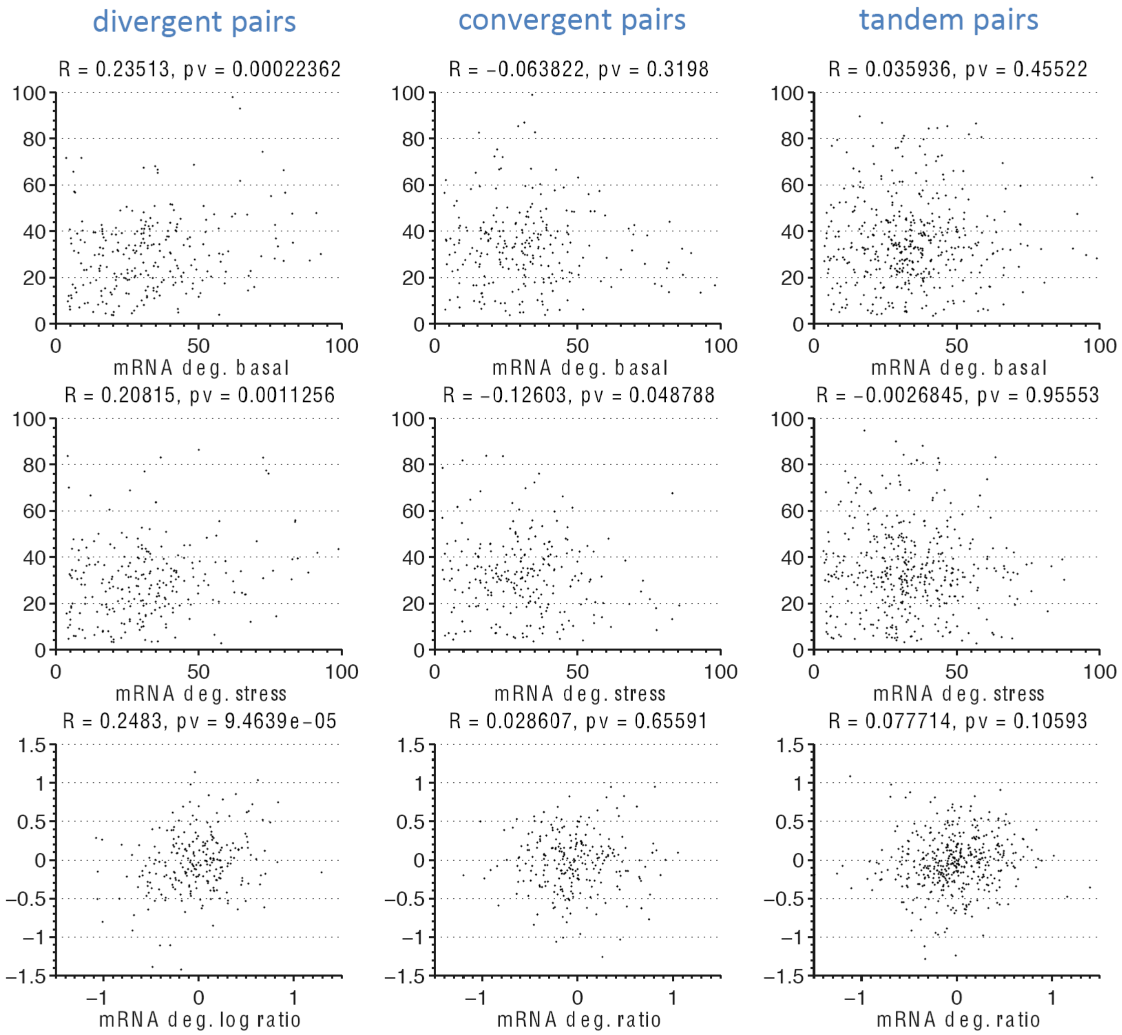
divergent pairs
convergent pairs
tandem pairs
R = 0.23513, pv = 0.00022362
R = −0.063822, pv = 0.3198
R = 0.035936, pv = 0.45522
mRNA deg. basal
R = 0.20815, pv = 0.0011256
R = −0.12603, pv = 0.048788
R = −0.0026845, pv = 0.95553
mRNA deg. stress
R = 0.2483, pv = 9.4639e−05
R = 0.028607, pv = 0.65591
R = 0.077714, pv = 0.10593
mRNA deg. log ratio
mRNA deg. ratio

Figure S4

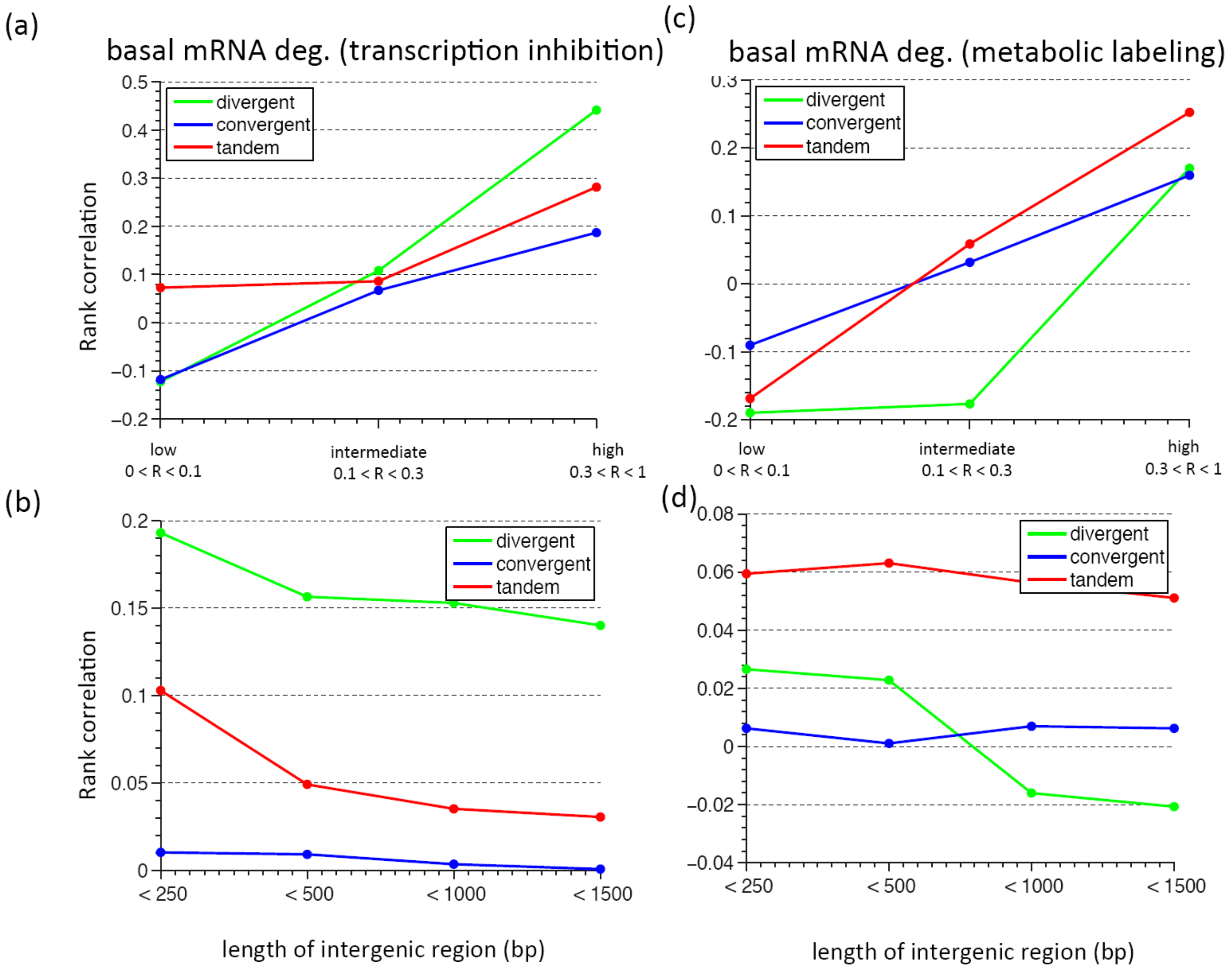
(a)
basal mRNA deg. (transcription inhibition)
(c)
basal mRNA deg. (metabolic labeling)
divergent
convergent
tandem
Rank correlation
low
0 < R < 0.1
intermediate
0.1 < R < 0.3
high
0.3 < R < 1
(b)
(d)
< 250
< 500
< 1000
< 1500
length of intergenic region (bp)

Figure S5

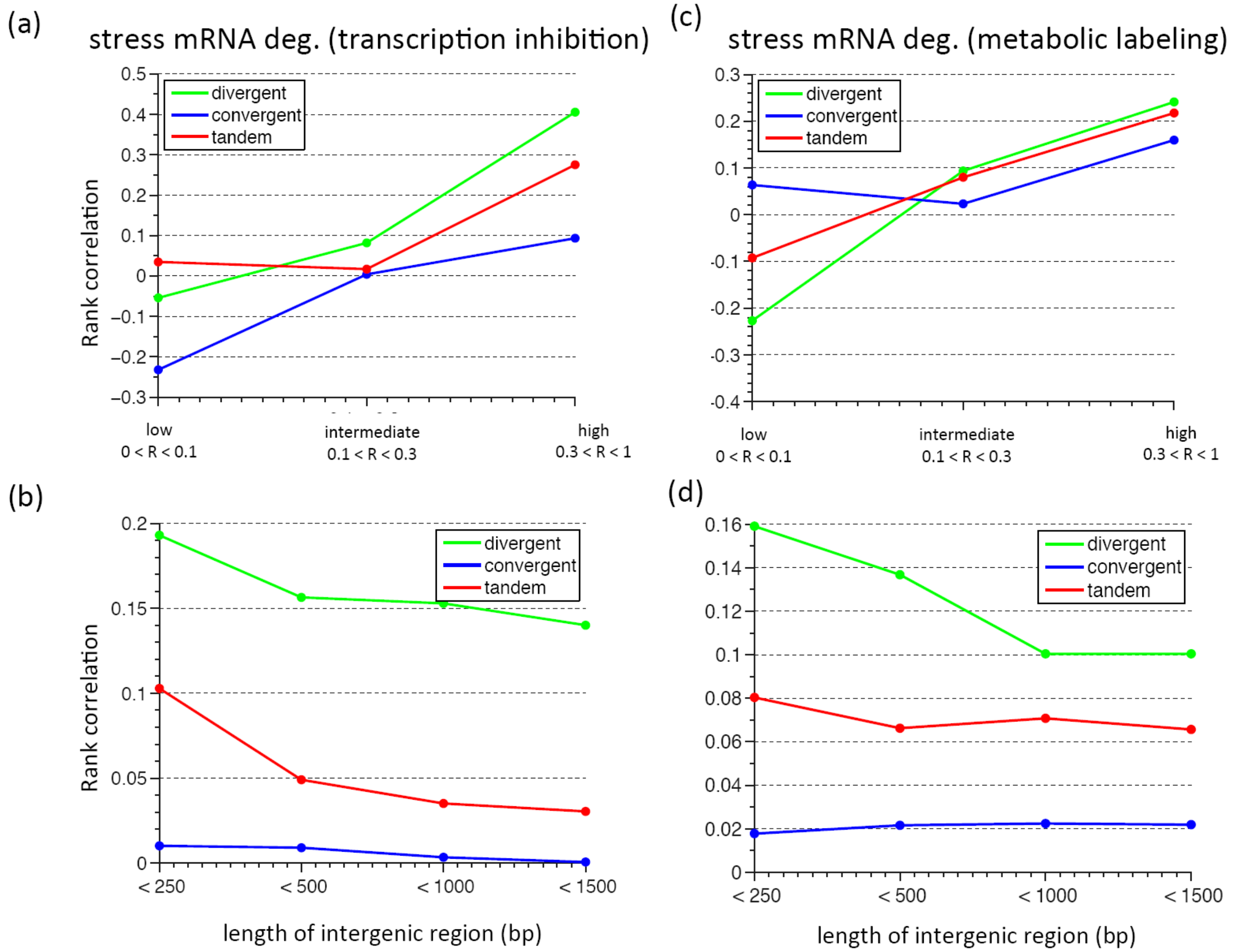
(a)
stress mRNA deg. (transcription inhibition)
(b)
(c)
stress mRNA deg. (metabolic labeling)
(d)
divergent
convergent
tandem
Rank correlation
low
0 < R < 0.1
intermediate
0.1 < R < 0.3
high
0.3 < R < 1
< 250
< 500
< 1000
< 1500
length of intergenic region (bp)

Figure S6

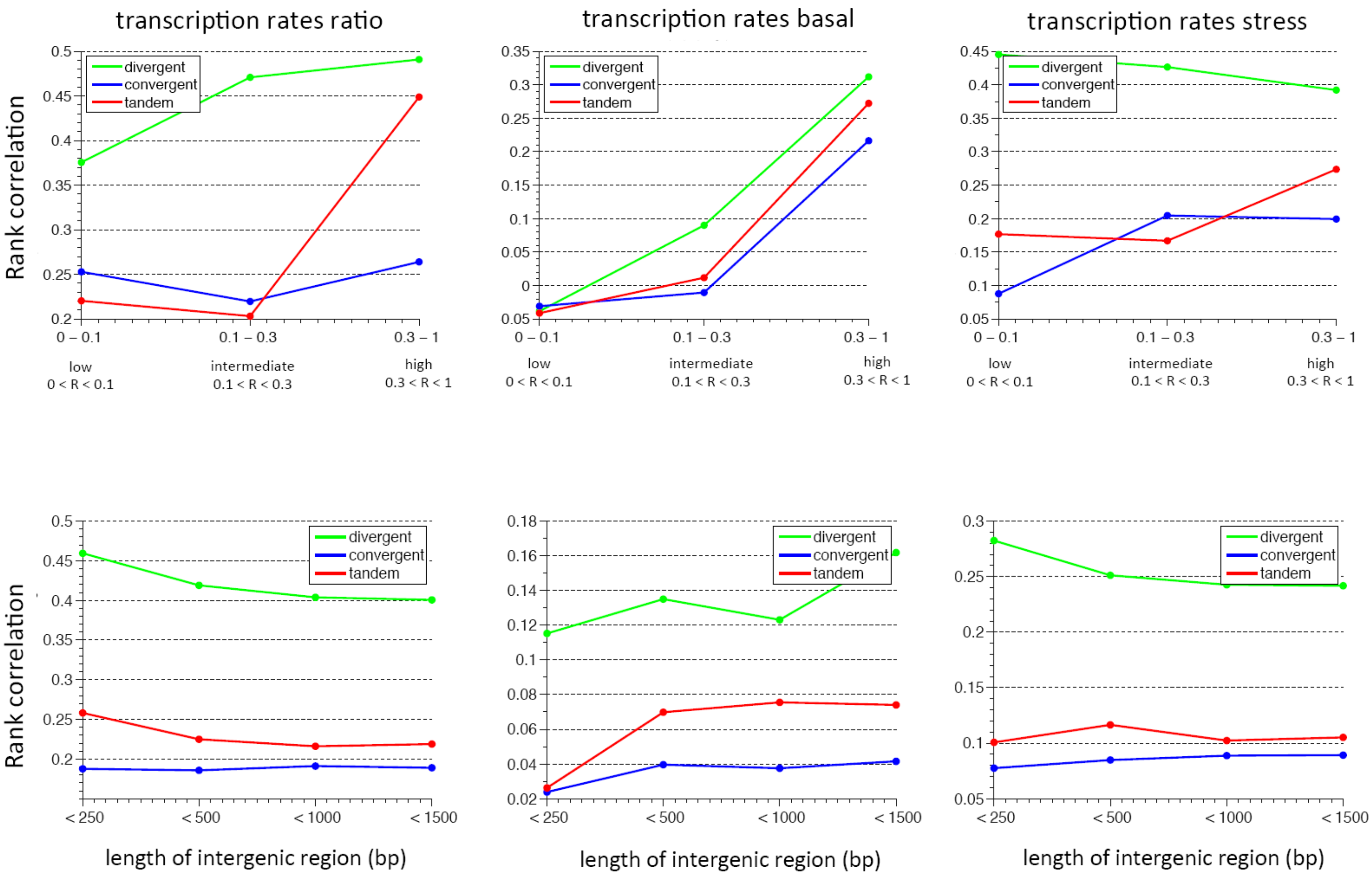

transcription rates ratio
transcription rates basal
transcription rates stress
Rank correlation
divergent
convergent
tandem
0 – 0.1
0.1 – 0.3
0.3 – 1
low
0 < R < 0.1
intermediate
0.1 < R < 0.3
high
0.3 < R < 1
< 250
< 500
< 1000
< 1500
length of intergenic region (bp)

Figure S7

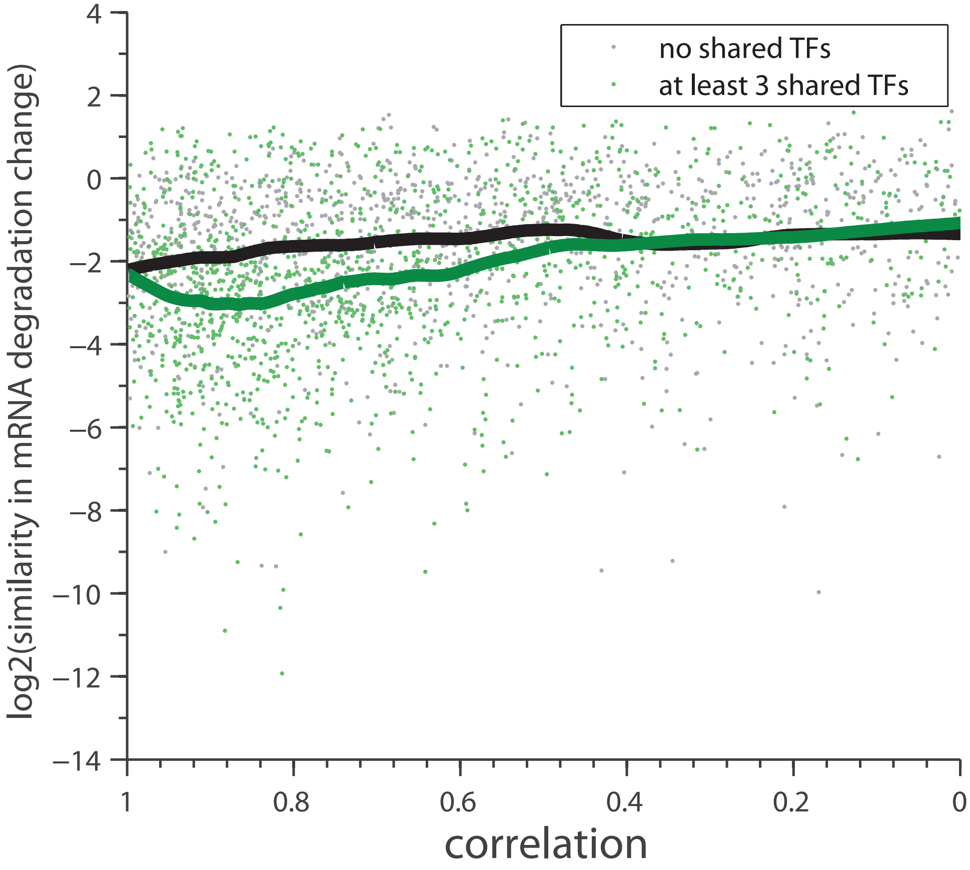

Figure S8

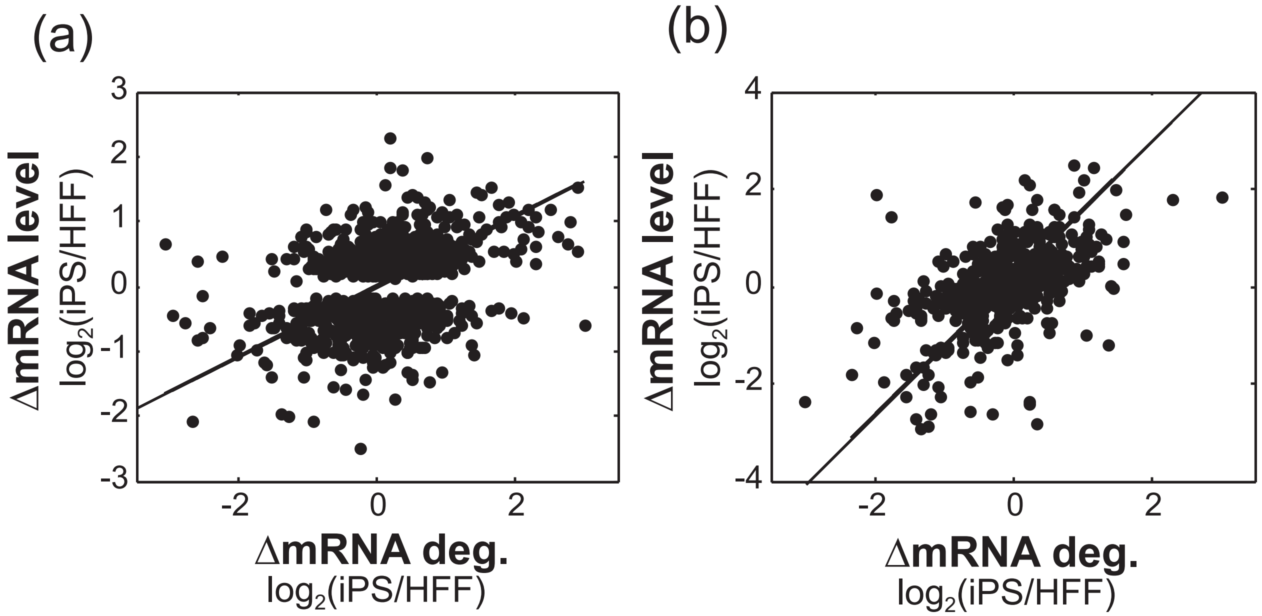

Figure S9

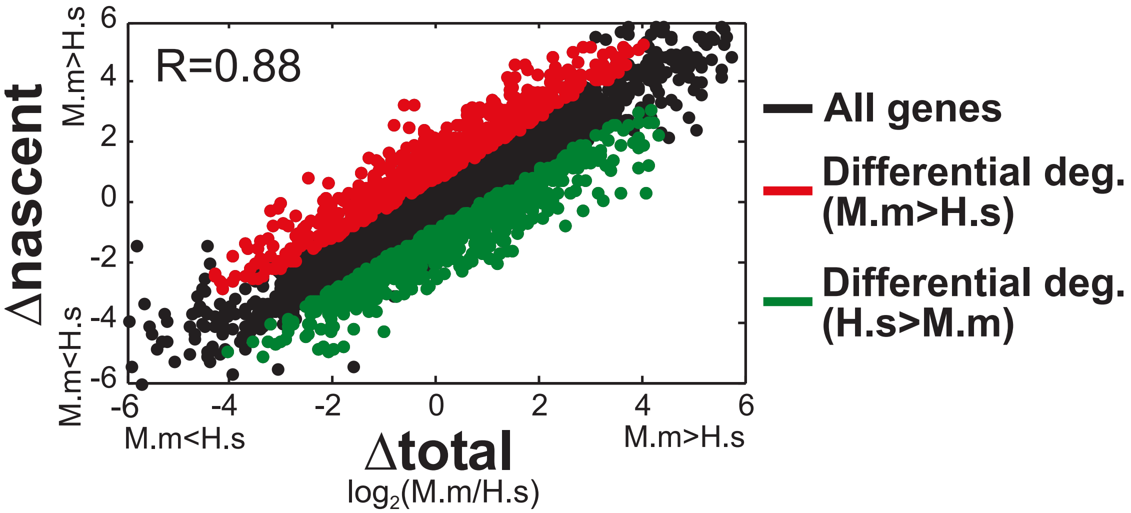

Figure S10

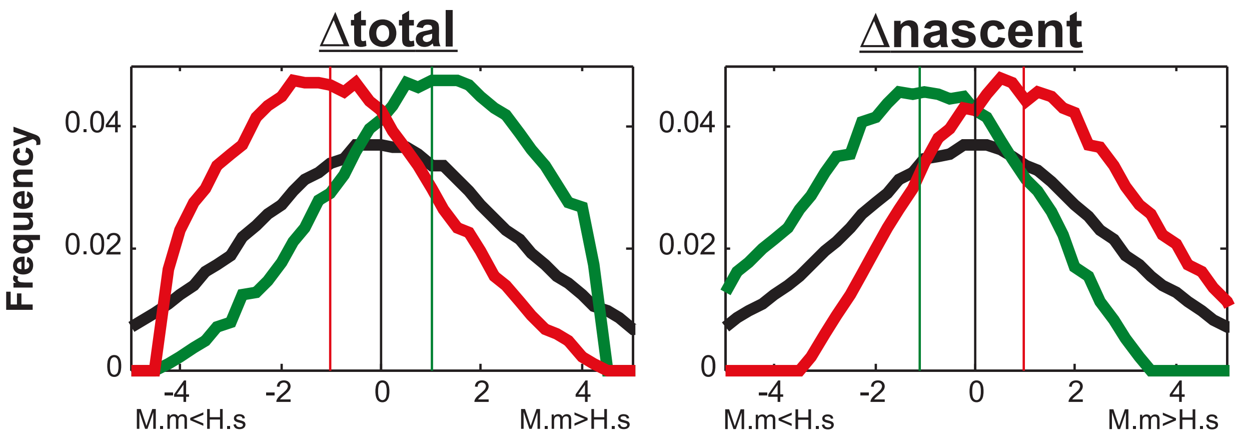

Figure S11
